# Management of diabetic ocular complications: from cellular insights to community strategies

**DOI:** 10.1186/s12886-024-03422-9

**Published:** 2024-04-10

**Authors:** Padmaja Kumari Rani

**Affiliations:** Department of Teleophthalmology, L V Prasad Eye Institute, 500034 Hyderabad, Telangana India

## Abstract

The editorial outlines an integrated approach to managing diabetic ocular complications, combining advanced scientific research with practical public health strategies to improve the prevention, diagnosis, and treatment of diabetic retinopathy and macular edema globally.

Global diabetes prevalence in 2017 encompassed an estimated 451 million adults, with projections indicating an increase to 693 million by 2045. A significant portion of these individuals remain undiagnosed (more than 50%), with significant numbers (more than 75%) in low- to middle-income countries [1]. Ocular complications, prevalent among people with diabetes, include refractive errors, cataracts, Diabetic Retinopathy (DR), glaucoma, retinal vein occlusions, and ischemic optic neuropathy. DR alone affects 22% of the diabetic population, with vision-threatening DR present in 6% of these individuals. Geographically, the prevalence of DR is notably high in Africa (35.90%) and North America with the Caribbean (33.30%), in stark contrast to South and Central America (13.37%) [2]. This editorial seeks to emphasize the critical need for a transition from cellular insights to community-level interventions in managing diabetic ocular complications.

Understanding the pathogenesis of diabetic retinopathy (DR) and diabetic macular edema (DME) has recently expanded beyond traditional pathways. Current research reveals that virtually all retinal cellular pathways contribute to the disease’s progression. This includes the breakdown of the blood-retinal barrier (BRB), drainage dysfunction in Müller glia and retinal pigment epithelium (RPE), and the combined effects of inflammation, oxidative stress, and neurodegeneration [3]. Such complexity necessitates a multifactorial approach to therapy and prevention.

Emerging evidence suggests the role of systemic factors in DR progression, with dietary interventions, systemic glycemic control, and the so-called ‘legacy effect’—the influence of historical glycemic control on future complication risks—being pivotal. The concept of metabolic memory, wherein previous hyperglycemic environments continue to affect cellular responses despite later glycemic control, raises the question of reversibility and further preventive strategies [4]. 

The gut-retina axis introduces unconventional avenues for managing DR, such as addressing gut dysbiosis, which may contribute to systemic inflammation and exacerbate retinal pathology. The therapeutic use of probiotics, drugs derived from microbial metabolites, and even fecal microbiota transplantation are being explored for their potential to modulate this axis and decelerate DR [5]. 

Moreover, genetic markers like MicroRNA-21 are gaining attention for their role in DR pathogenesis, offering potential targets for gene-based interventions [6]. Advances in imaging, especially with noninvasive technologies like wide-angle OCT angiography, provide valuable insights into ischemia quantification and DR severity, which can guide treatment decisions and follow-up care [7]. Wide-angle OCT angiography-based classification has the potential to shift the treatment paradigm in diabetic retinopathy.

Novel therapeutic agents targeting the Angiopoietin-2 (Ang-2) and Vascular Endothelial Growth Factor (VEGF) pathways have initially shown significant efficacy in treating age-related macular degeneration and are now promising similarly effective interventions for managing the sight-threatening complications associated with DR [8]. This therapeutic evolution underscores the necessity of a nuanced understanding of cellular mechanisms to strategically combat the disease.

The translation of cellular insights into community strategies requires a nuanced, multifaceted approach. There’s no one-size-fits-all solution for managing diabetic ocular complications. Community initiatives should include targeted screenings such as those offered by comprehensive diabetic care centers and diabetes clinics, opportunistic screenings at workplaces, training for first point-of-care teams like optometrists in diabetic retinopathy (DR) screening and referral guidelines, awareness and education programs for individuals with diabetes, and the use of point-of-care technologies, such as fundus cameras, for diabetic retinopathy screening.

Improving image quality is pivotal for enhancing Artificial Intelligence (AI) model accuracy, which benefits from the integration of human intelligence, like trained fundus photographers skilled in DR grading [9]. Mydriatic fundus photography can enhance image gradability, particularly in older individuals with cataracts. In a study of nonmydriatic fundus imaging within a rural diabetic eye care model, the gradability of posterior segment images was limited to 62%. Factors contributing to ungradable images included small pupils, defocused or incomplete fields of view, and cataracts [10]. For improved AI accuracy in diabetic retinopathy (DR) screening, mydriatic fundus photography is crucial, especially beneficial for those over 50 or with visual acuity below 6/12. Studies show that, using mydriasis, the proportion of non-gradable images in a rural screening model dropped from 29.1 to 8.6% [11]. Despite concerns, the risk of angle closure glaucoma post-mydriasis was found to be minimal [12]. Thus, mydriatic imaging could be implemented to improve the gradability of images in diabetic retinopathy screenings.

Effective AI triage systems, physician-led DR screening models, and general ophthalmologist-DR management training programs are integral to this strategy. Additionally, the utilization of mobile units equipped with fundus cameras, OCT, and lasers extends the reach of these services.

Teleophthalmology linkages are critical, facilitating coordination between primary care physicians and ophthalmologists and establishing horizontal and vertical connections through the healthcare system. This comprehensive ‘cell to community’ model ensures that advanced scientific discoveries are translated into practical, accessible care.

Conclusively, the ‘cell to community’ model epitomizes a holistic strategy for diabetic eye disease management. By unraveling the cellular underpinnings of DR and DME, we pave the way for tailored therapies that tackle the disease’s genetic and molecular roots. Simultaneously, adaptive community strategies, underpinned by technological advancements and professional expertise, are essential to enhancing diagnostic and therapeutic accessibility. Bridging cutting-edge research with pragmatic public health policies is imperative to mitigate the global impact of diabetic ocular complications. The synergy of microscopic understanding and macroscopic action holds the key to safeguarding vision for people with diabetes worldwide.

## Data Availability

No datasets were generated or analysed during the current study.
